# Clinical features and related factors of freezing of gait in patients with Parkinson's disease

**DOI:** 10.1002/brb3.2359

**Published:** 2021-09-22

**Authors:** Fengting Zhang, Jin Shi, Yangyang Duan, Jiang Cheng, Hui Li, Tingting Xuan, Yue Lv, Peng Wang, Haining Li

**Affiliations:** ^1^ School of Clinical Medicine Ningxia Medical University Yinchuan China; ^2^ Department of Neurology General Hospital of Ningxia Medical University Ningxia Key Laboratory of Cerebrocranial Diseases Incubation Base of National Key Laboratory Yinchuan China; ^3^ Department of Computer Science Jiangsu Ocean University Lianyungang China

**Keywords:** freezing of gait, new gait freezing questionnaire, Parkinson's disease, related factors

## Abstract

**Background:**

Freezing of gait (FOG) is a disabling paroxysmal gait disorder that prevents starting or resuming walking, which seriously negatively affects patients’ quality of life (QOL). The diagnosis and treatment of FOG remain a huge medical challenge. The purpose of this study was to explore the clinical characteristics and related factors of FOG in patients with Parkinson's disease (PD).

**Methods:**

The motor and nonmotor symptoms of a total number of 77 PD patients were evaluated. Patients with or without FOG were defined as a score ≥1 in the new freezing of gait questionnaire (NFOG‐Q). A comparative study between patients with and without FOG was conducted.

**Results:**

In this investigation, the prevalence of FOG was 48%. The patients with FOG had longer disease duration, higher Hoehn‐Yahr stage (H‐Y stage), higher dose of dopaminergic agents, and higher nonmotor and motor symptom scores. A significant positive correlation was found between the NFOG‐Q score and the H‐Y stage, PIGD subscore, PDQ‐39, and the attention/memory in the nonmotor symptoms assessment scale (NMSS) subitem (*r* > 0.5, *p* < .05). The binary logistic regression analysis showed that the higher H‐Y stage, higher rigidity subscore and Unified Parkinson's Disease Rating Scale II (UPDRS II) score, and UPDRS III score were significantly correlated with the occurrence of FOG (*p* < .05). In the analysis of the frequency of FOG, the prevalence of FOG in H‐Y stage was higher in the middle and late stages, and the prevalence of FOG increased with the increase in PDQ‐39 scores.

**Conclusion:**

The severity of FOG was significantly positively correlated with the H‐Y stage, PIGD subscore, PDQ‐39 score, and attention/memory score. Based on our findings, we conclude that the clinical characteristics of rigidity can be used as a potential predictor of FOG.

## Graphical abstract

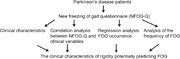

## 1 INTRODUCTION

Freezing of gait (FOG) is defined as a temporary loss or reduction in the movement of the foot forward despite the intention to walk (Nutt et al., 2011), a sudden feeling that one's foot seems to be stuck to the floor. Under normal circumstances, the symptoms last for a few seconds, occasionally more than 30 s, but sometimes that inability to produce effective steps continues even longer (Schaafsma et al., 2003). The pathophysiological mechanism of FOG is still unclear despite the extensive research that has been conducted to date. This phenomenon is characterized by suddenness and heterogeneity, as well as by a wide range of cognitive, behavioral, and environmental factors. The interactions between the vulnerability of the motor network and the regulatory factors lead to a failure of neuronal integration (Daniel et al., 2019) and clinical manifestations of FOG (Lewis & Shine, 2016). Considering the intermittent and unpredictable nature of FOG, a recent study (Demrozi et al., 2020) claimed for the first time that a wearable system can be used to identify the pre‐stage of FOG patients to predict FOG occurrence. The average device sensitivity and specificity can reach 94.1% and 97.1%, and FOG occurrence can be monitored in real time. Thus, the prediction and identification of FOG have been greatly improved by this invention. The new freezing of gait questionnaire (NFOG‐Q) is a reliable nondetection system for retrospective detection and follow‐up of FOG, whose potential for reliable evaluation of FOG has been confirmed (Nieuwboer et al., 2009). FOG is common in patients with a later period in Parkinson's disease (PD), and approximately 50% of the patients are affected by that condition (Giladi et al., 2009). Many associated factors are to be considered in FOG, including motor symptoms, mood, cognition, and the environment. Previous studies have reported that FOG is associated with longer disease duration, a higher Unified Parkinson's Disease Rating Scale (UPDRS) score, a higher daily dose of levodopa, and previous exposure to anticholinergic drugs (Perez‐Lloret et al., 2014). In a meta‐analysis, FOG was found to be related to the severity and duration of the disease (Ge et al., 2020). FOG was also correlated with bradykinesia (Aktürk et al., 2019). In terms of nonmotor symptoms, FOG patients manifest more pronounced anxiety than those without FOG (Martens et al., 2016); moreover, the former are more likely to have cardiovascular, gastrointestinal, and urinary symptoms (Choi et al., 2018). Prolonged saccade latency could be a practical oculomotor parameter that can be used for the identification of FOG development and its progression (Wu et al., 2020). According to the UPDRS scores used for the assessment of the severity of PD, the severity of the subitems of tremor, rigidity, bradykinesia, posture, and postural instability gait difficulty (PIGD) were evaluated. This Hoehn‐Yahr stage (H‐Y stage) is used to assess the disease stage and it is divided into early, middle, and late stages. We utilized PD questionnaires‐39 (PDQ‐39), consisting of 39 questions (eight dimensions), which can reflect the quality of life (QOL) of PD patients in the past 1 month and evaluate the QOL of patients.

In the present study, we elucidated the clinical characteristics of FOG in PD and established the associated influencing factors, which will facilitate the implementation of further research on the early intervention and pathogenesis of FOG, and will contribute to the improvement of patients’ QOL.

## 2 MATERIALS AND METHODS

### 2.1 Participants

The study population included 77 patients with PD that were recruited from the Department of Neurology of the General Hospital of Ningxia Medical University (Yinchuan, Ningxia, China). All patients were diagnosed based on the UK Parkinson's Disease Brain Bank Criteria (Hughes et al., 1992). The following exclusion criteria were applied: neurological diseases other than standard PD, atypical or secondary PD, dementia, deep brain stimulation (DBS) surgery, severe mental or systemic diseases, such as those of the heart, liver, or kidneys, and clinical evaluation failure. All patients provided written informed consent. All protocols of this study were approved by the Human Body Research Institution Review Committee of Ningxia Medical University (2019‐160).

### 2.2 Statistical analysis

All statistical analyses were performed with SPSS, version 22.0 for Windows (IBM, Armonk, NY, USA), and all graphs were developed using GraphPad Prism 7.0 (GraphPad Software Inc., San Diego, CA, USA). Continuous variables were expressed as mean ± SD or median (25th percentile, 75th percentile) whereas categorical variables as percentages (%). Student's *t*‐test was used for continuous variables which conformed to the normal, and Mann–Whitney U‐test was applied those that did not conform to the normal distribution or level variable. The χ^2^ test was used for categorical variables, and Pearson correlation analysis was conducted to analyze the correlation between NFOG‐Q score and clinical variables. Binary logistic regression was implemented to assess the influencing factors associated with FOG. Statistical significance was defined at *p* < .05.

### 2.3 Clinical assessment and groups

The general data of the patients were collected, including gender, age, age at disease onset, education level, past history, side of symptoms at disease onset, past and present oral drugs received, and results of the physical examination of the nervous system. A comparative study was then conducted between the two groups.

NFOG‐Q was used to ask whether a sudden feeling of feet glued to the floor was experienced when a step was initiated or during walking or turning. The researcher imitated the symptoms of FOG in front of each patient, so that they could better understand the manifestations of FOG. NFOG‐Q ≥1 point was defined as the presence of PD with FOG.

Standardized scales were used for clinical evaluation of various parts, including the UPDRS scores to assess the severity of PD and the H‐Y stage to assess the disease stage. Our nonmovement symptom scale assessment included the utilization of PDQ‐39, the nonmotor symptoms assessment scale (NMSS) and subitem, the Montreal Cognitive Assessment (MoCA), the Hamilton Anxiety Scale (HAM‐A), the Hamilton Depression Scale (HAMD), the Epworth Sleepiness Scale (ESS), the Department of Psychiatry to The Chinese University of Hong Kong Sleep Questionnaire (RBD‐SQ), and the Autonomic Nerve function Scale (SCPA‐AUT). The UPDRS severity of tremor (items 16, 20, and 21), rigidity (item 22), bradykinesia (items 23−26, 31), PIGD subscore (items 27−30) were further investigated. The patients were examined in the “on” condition.

## 3 RESULTS

### 3.1 Clinical characteristics of PD with FOG and PD without FOG

The clinical characteristics of the two groups are presented in Table 1. Of the 77 patients with PD, 37 patients with FOG (48%) were included. The statistical analysis of the general clinical data revealed no statistical differences between the two groups in gender, age, age of onset, education level, and first‐onset side. PD patients with FOG had a longer disease duration and were taking a higher dose of dopaminergic agents than those without FOG (*p* < .01). The motor symptom assessment showed higher H‐Y stage, severity of rigidity, bradykinesia, PIGD, and scores of each part of UPDRS in the patients with FOG than in those without FOG (*p* < .05). No statistically significant difference was established in the tremor subscore between the two groups. The nonmotor symptom assessment found higher PDQ‐39, NMSS total, HAM‐A, HAMD, ESS, and SCPA‐AUT in the patients with FOG than in the those without FOG (*p* < .05). No statistical difference was observed between the two groups in MoCA, Argentina hyposmia rating scale (AHRS), and RBD‐SQ.

**TABLE 1:** Clinical characteristics of Parkinson's disease (PD) with freezing of gait (FOG) and PD without FOG

	PD without FOG (*n* = 40)	PD with FOG (*n* = 37)	*p*‐Value
Sex (male/female)	21/19	19/18	.920
Age (years)	60.83 ± 8.58	63.92 ± 10.52	.160
Age at disease onset (years)	56.05 ± 8.54	56.31 ± 10.93	.907
Duration of disease (years)	4.5 (2.0,5.0)	7.0n(4.0,10.0)	<.001
Education level (years)	7.08 ± 4.03	7.14 ± 3.77	.112
H‐Y stage	2.30 ± 0.62	3.18 ± 0.60	<.001
Side of symptoms at disease onset			.232
Right	13 (32.5)	12 (32.4)	
Left	22 (55.0)	15 (40.5)	
Bilateral	5 (12.5)	10 (27.0)	
Levodopa equivalent dose (mg)	402.55 ± 171.10	545.9 ± 218.64	.002
Tremor subscore	8.85 ± 5.12	10.11 ± 6.45	.344
Rigidity subscore	5.78 ± 2.61	8.95 ± 3.37	<.001
Bradykinesia subscore	11.33 ± 5.36	17.08 ± 6.60	<.001
PIGD subscore	3.65 ± 2.17	5.86 ± 2.42	<.001
UPDRS part I score	4.33 ± 3.25	6.46 ± 3.91	.011
UPDRS part II score	12.70 ± 5.74	23.32 ± 8.50	<.001
UPDRS part III score	30.08 ± 12.41	41.57 ± 15.38	.001
UPDRS part IV score	3.15 ± 2.00	6.62 ± 4.17	<.001
PDQ‐39 score	43.68 ± 26.02	70.43 ± 32.44	<.001
NMSS score	65.43 ± 30.79	89.41 ± 34.46	.002
MoCA score	20.90 ± 6.20	19.14 ± 6.79	.237
HAM‐A score	12.33 ± 8.22	18.78 ± 9.34	.002
HAMD score	13.33 ± 9.59	20.71 ± 11.02	.002
ESS score	7.40 ± 5.95	10.89 ± 5.99	.009
AHRS score	19.08 ± 5.77	16.86 ± 8.64	.188
RBD score	28.53 ± 28.47	30.03 ± 24.39	.805
SCPA‐AUT score	31.80 ± 6.97	35.87 ± 9.88	.039
NFOG‐Q score	‐	21.35 ± 6.36	‐

Abbreviations: ESS, Epworth Sleepiness Scale; H‐Y stage, Hoehn‐Yahr stage; HAM‐A, Hamilton Anxiety Scale; HAMD, Hamilton Depression Scale; NMSS, nonmotor symptoms assessment scale; MoCA, Montreal Cognitive Assessment; PDQ‐39, Parkinson's disease questionnaires‐39; PIGD, postural instability gait difficulty; UPDRS, Unified Parkinson's Disease Rating Scale.

### 3.2 Correlation analysis between NFOG‐Q and clinical variables

No significant correlation was established between the NFOG‐Q score and age, age at disease onset and duration of disease, whereas a statistically significant positive correlation was detected between the NFOG‐Q score and the H‐Y stage (*r* = 0.597, *p* < .0001). Additionally, the NFOG‐Q score and the PIGD subscore were significantly positively associated (*r* = 0.639, *p* < .0001).

The analysis of the correlation between the NFOG‐Q score and the clinical variables of the nonmotor symptoms showed that the NFOG‐Q and PDQ‐39 scores (*r *= 0.624, *p* < .001) were significantly positively correlated (Table 2). The analysis of the correlation between the NFOG‐Q score and the NMSS subitem clinical variables showed a significant positive correlation between NFOG‐Q and the attention/memory score (Table 3).

**TABLE 2:** Association between freezing of gait questionnaire (FOG‐Q) and clinical variables in patients with freezing of gait (FOG)

	NFOG‐Q	
	Pearson's correlation (*γ*)	*p‐*Value
Age (years)	0.149	.379
Age at disease onset (years)	0.144	.392
Duration of disease (years)	0.089	.598
Levodopa equivalent dose (mg)	−0.040	.816
H‐Y stage	0.597	<.0001
Tremor subscore	0.032	.850
Rigidity subscore	0.164	.333
Bradykinesia subscore	0.413	.0111
PIGD subscore	0.639	<.0001
UPDRS part I score	0.495	.002
UPDRS part II score	0.233	.0007
UPDRS part III score	0.405	.0129
UPDRS part IV score	0.496	.0018
PDQ‐39 score	0.624	<.001
NMSS score	0.356	.0307
MoCA score	0.450	.0052
HAM‐A score	0.369	.0244
HAMD score	0.451	.0051
ESS score	0.198	.239
AHRS score	−0.034	.842
RBD‐SQ score	0.381	.0199
SCPA‐AUT score	0.158	.349

Abbreviations: ESS, Epworth Sleepiness Scale; H‐Y stage, Hoehn‐Yahr stage; HAM‐A, Hamilton Anxiety Scale; HAMD, Hamilton Depression Scale; NMSS, nonmotor symptoms assessment scale; MoCA, Montreal Cognitive Assessment; PDQ‐39, Parkinson's disease questionnaires‐39; PIGD, postural instability gait difficulty; UPDRS, Unified Parkinson's Disease Rating Scale.

**TABLE 3:** Correlation analysis between new freezing of gait questionnaire (NFOG‐Q) score and nonmotor symptoms assessment scale (NMSS) subitems

	NFOG‐Q	
	Pearson's correlation (γ)	*p‐*Value
Cardiovascular score	0.365	.026
Sleep/fatigue score	0.310	.062
Mood/apathy score	0.488	.002
Perceptual/hallucinations score	0.199	.238
Attention/memory score	0.514	.001
Gastrointestinal tract score	0.383	.019
Urinary score	0.111	.513
Sexual function score	0.089	.599
Miscellaneous score	0.164	.233

### 3.3 Regression analysis of the factors affecting FOG occurrence

The binary logistic regression analysis indicated that a higher H‐Y stage (*p* = .006), rigidity subscore (*p* = .005), UPDRS II score (*p* = .002), and UPDRS III score (*p* = .008) were significantly associated with the occurrence of FOG (Table 4).

**TABLE 4:** Binary logistic regression analysis of the influencing factors of freezing of gait (FOG)

	OR (95%CI)	*p‐*Value
H‐Y stage	42.163 (2.985–595.526)	.006
Rigidity subscore	1.580 (0.694–0.947)	.005
UPDRS II	1.498 (1.159–1.937)	.002
UPDRS III	0.811 (0.694–0.947)	.008

Abbreviations: H‐Y stage, Hoehn‐Yahr stage; UPDRS, Unified Parkinson's Disease Rating Scale.

### 3.4 Analysis of the frequency of FOG

**FIGURE 1:**
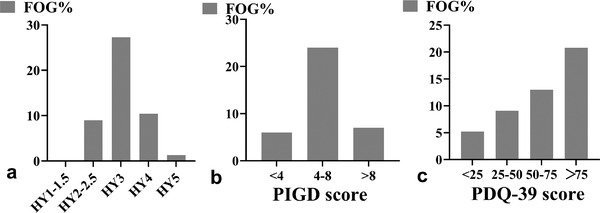
The frequency of freezing of gait (FOG) across Hoehn‐Yahr stage (H‐Y) stages, in different postural instability gait difficulty (PIGD) scores, and Parkinson's disease questionnaires‐39 (PDQ‐39) scores

The frequency of FOG across H‐Y stages, in different PIGD scores, and PDQ‐39 scores are presented in Figure 1. The prevalence of FOG in H‐Y stage 1.0−5.0 was 0%, 9.01%, 27.27%, 10.39%, and 1.30%. The mid‐term prevalence of FOG was the highest (Figure 1a). The prevalence of FOG in PIGD subscores <4 scores, 4–8 scores, and >8 scores were 10.39%, 40.26%, and 11.69% (Figure 1b). The prevalence of FOG in PDQ‐39 scores of <25 scores, 25–50 scores, 50–75 scores, and >75 scores was5.19%, 5.09%, 12.99%, and 20.78%, respectively. The prevalence rate gradually increased with the rise in the PDQ‐39 scores (Figure 1c).

## 4 DISCUSSION

In our study, the incidence of PD with FOG was 48%, which is consistent with a previous report showing that FOG was common in patients with a later PD stage and that approximately 50% of the patients were affected (Giladi et al., 2009). Importantly, the incidence of FOG increases with the prolongation of the disease. Furthermore, patients with FOG have higher H‐Y grades, higher UPDRS scores, rigidity, and higher bradykinesia and PIGD subscores. A significantly positive correlation was found between the NFOG‐Q score and the H‐Y stage, the PIGD subscore, PDQ‐39, and the attention/memory in the NMSS subitem. In the binary logistic regression analysis, the H‐Y stage, UPDRS II, UPDRS III, and rigidity subscore were found to be significant influencing factors for the occurrence of FOG. The prevalence of FOG in H‐Y stage was higher in the middle and late stages, and the prevalence of FOG increased with the rise in the PDQ‐39 scores. These results are identical to the findings of other research that established associations between FOG and longer disease course and higher UPDRS scores (Ge et al., 2020; Perez‐Lloret et al., 2014). The findings presented are consistent with previous reports that stiffness, motor retardation, postural instability, and high speech disorder scores in patients with PD are risk factors for the occurrence of FOG (Avanzino et al., 2018; Zhang et al., 2016). In a 12‐year follow‐up study of patients with PD, the severity of PIGD symptoms and psychiatric symptoms were independently related to FOG (Forsaa et al., 2015). In our study, no difference was found between the patients with and without FOG in tremor severity. The PIGD subtype of PD is considered to be more common in patients with FOG than the tremor‐dominated subtype of PD (Vervoort et al., 2016). Other research found no relationship between retardation and FOG and considered that FOG is an independent symptom (Contreras & Grandas, 2012). However, most results suggest that rigidity, bradykinesia, and PIGD severity were associated with the occurrence of FOG (Avanzino et al., 2018; Forsaa et al., 2015; Vervoort et al., 2016; Zhang et al., 2016).

Concerning emotional disorders, patients with FOG were more anxious than those without FOG (Martens et al., 2016). Patients were more likely to experience FOG when walking with anxiety (Ehgoetz et al., 2014). Regarding the pathophysiological mechanism of its occurrence, evidence has shown that competitive and complementary relationships occurring among the motor, cognitive, and limbic circuits in the case of overload in the basal ganglia hamper the proper movement and lead to the occurrence of FOG (Lewis & Barker, 2009). We analyzed for the first time the correlation between FOG and anxiety/attention. A higher anxiety level was related to a longer time required to switch attention between different tasks (Martens et al., 2016). Our research results also showed that the severity of FOG was significantly positively correlated with the severity of attention/memory. Nevertheless, it is difficult to infer the existence of a causal relationship between anxiety and FOG and whether FOG precedes anxiety, or anxiety leads to FOG. Although, drug or cognitive behavioral therapy can be administered to treat anxiety in the early stage of PD gait disorder, the side effects of drugs will affect FOG and its association with mood disorder. Therefore, for achieving optimal results, a combination of psychotherapy and physiotherapy needs to be applied at an early stage. Anxiety and depression often coexist, and treating anxiety can improve depression.

As a paroxysmal gait disorder, FOG can easily increase the risk of fall events during daily activities, greatly affecting the QOL of patients (Walton et al., 2015). Previously, gait problems were established to be an independent factor associated with poor QOL in the COPPADIS cohort of patients with FOG (Santos et al., 2019). Our research findings show that the severity of FOG is significantly positively correlated with the decline in QOL. Therefore, more attention is to be paid to improve patients’ ability to perform daily life activities. Physiotherapy is gradually emerging as therapeutic approach for FOG treatment. A recent study carried out antigravity treadmill training to improve the daily life activity ability of FOG patients (Baizabal‐Carvallo et al., 2020). In another examination of FOG motor networks, a novel device for deep brain electrical stimulation was used, which recorded the neural activity of the subthalamic nucleus. The authors found that FOG was related to a disorder of the cortex‐subthalamic nucleus network (Valentino et al., 2014). Therefore, new treatment strategies (such as neuromodulation techniques) are required for the treatment of patients with FOG. Although neurostimulation is promising, it may require the application of invasive and expensive technologies. In the future, physical training therapy combined with a real‐time prediction system can be used for family‐based assessment and optimization of a comprehensive drug therapy, as well as for the timely detection of psychological and physical signs of nonmotor symptoms.

We found no difference in ESS severity with or without FOG. Studies have shown that the degradation of the locus coeruleus and the ascending reticular activating system are related to the development of excessive daytime sleepiness (Banks et al., 2019). Noradrenergic deficits owing to locus coeruleus cell loss have been interrelated with poor balance, falls, and possibly FOG. The severity of RBD‐SQ in patients with FOG was not different from that of those without FOG. Consistent with previous studies, although gait disorders were found in RBD patients diagnosed with polysomnography (Alibiglou et al., 2016; Ehgoetz et al., 2019), we found no statistically significant difference between the RBD‐SQ severity in patients with FOG and those without FOG (Banks et al., 2019; Ehgoetz et al., 2018). The cognitive dysfunction was identified as an independent risk factor for FOG (Forsaa et al., 2015), and no significant differences were established between patients with FOG and the those without FOG in the cognitive function after adjustments for covariates and disease severity (Morris et al., 2020). In our study, no statistically significant difference was observed in the cognitive function between patients with FOG and those without FOG. Nevertheless, differences in the cognitive function of such groups of patients need to be further investigated. Our findings show that the decreased sense of smell was not correlated with the severity of FOG, nor was it a risk factor affecting the occurrence of FOG. An earlier study established that patients with lower education levels were more likely to have FOG. This outcome might have occurred because highly educated patients have a better understanding of PD and PD with FOG, which results in a better compliance with the prescribed treatment (Forsaa et al., 2015). However, in our study, we did not find differences between the education levels of the patients with FOG and those without FOG, probably due to the small sample size.

Certain limitations of our study are to be acknowledged. First, the evaluation of FOG was based mainly on scale evaluation results, most of which were reported by patient recall. Therefore, a recall bias might have affected the results of our study. Second, our study was cross‐sectional, with relatively few objects. In the future, a real‐time monitoring system is should be applied to evaluate FOG in patients; research on related biochemical markers can also be conducted.

## 5 CONCLUSION

In this study, we found that the severity of FOG was significantly positively correlated with the H‐Y stage, PIGD subscore, PDQ‐39 score, and attention/memory score. The clinical characteristics of rigidity can be used as potential predictors of FOG in PD. Furthermore, the severity of motor and nonmotor symptoms and decreased ability of daily living of FOG should be properly and timely addressed.

## CONFLICT OF INTEREST

The authors declare no conflict of interest.

## AUTHOR CONTRIBUTIONS

The work described here was done in mutual assistance with all the authors. Haining Li conducted research design, reviewed, and edited the manuscript. Peng Wang collected references and revised the manuscript. Fengting Zhang completed the data collection, performed statistical analysis, and drafted the manuscript. Yangyang Duan and Jin Shi conducted data collection. All the authors read and approved the final version of the manuscript.

### PEER REVIEW

The peer review history for this article is available at https://publons.com/publon/10.1002/brb3.2359

## Data Availability

The datasets used and analyzed in the current study are available from the corresponding author on reasonable request.
